# Hepatocellular carcinoma incidence at national and provincial levels in Iran from 2000 to 2016: A meta-regression analysis

**DOI:** 10.1371/journal.pone.0245468

**Published:** 2021-01-22

**Authors:** Nima Fattahi, Negar Rezaei, Mohsen Asadi-Lari, Moein Yousefi, Zahra Madadi, Kimiya Gohari, Ali Sheidaei, Esmaeil Mohammadi, Nazila Rezaei, Mahboubeh Parsaeian, Farzad Kompani, Farshad Farzadfar

**Affiliations:** 1 Non-Communicable Diseases Research Center, Endocrinology and Metabolism Population Sciences Institute, Tehran University of Medical Sciences, Tehran, Iran; 2 Endocrinology and Metabolism Research Center, Endocrinology and Metabolism Clinical Sciences Institute, Tehran University of Medical Sciences, Tehran, Iran; 3 Department of Epidemiology, School of Public Health, Iran University of Medical Sciences, Tehran, Iran; 4 Oncopathology Research Centre, Iran University of Medical Sciences, Tehran, Iran; 5 Department of Biostatistics, Faculty of Medical Sciences, Tarbiat Modares University, Tehran, Iran; 6 Department of Epidemiology and Biostatistics, School of Public Health, Tehran University of Medical Sciences, Tehran, Iran; 7 Division of Hematology and Oncology, Children's Medical Center, Pediatrics Center of Excellence, Tehran University of Medical Sciences, Tehran, Iran; University of Central Florida, UNITED STATES

## Abstract

**Background:**

The incidence of Hepatocellular carcinoma (HCC), the most common primary liver cancer with high mortality, is undergoing global change due to evolving risk factor profiles. We aimed to describe the epidemiologic incidence of HCC in Iran by sex, age, and geographical distribution from 2000 to 2016.

**Methods:**

We used the Iran Cancer Registry to extract cancer incidence data and applied several statistical procedures to overcome the dataset’s incompleteness and misclassifications. Using Spatio-temporal and random intercept mixed effect models, we imputed missing values for cancer incidence by sex, age, province, and year. Besides, we addressed case duplicates and geographical misalignments in the data.

**Results:**

Age-standardized incidence rate (ASIR) increased 1.17 times from 0.57 (95% UI: 0.37–0.78) per 100,000 population in 2000 to 0.67 (0.50–0.85) in 2016. It had a 21.8% total percentage change increase during this time, with a 1.28 annual percentage change in both sexes. Male to female ASIR ratio was 1.51 in 2000 and 1.57 in 2016. Overall, after the age of 50 years, HCC incidence increased dramatically with age and increased from 1.19 (0.98–1.40) in the 50–55 age group to 6.65 (5.45–7.78) in the >85 age group. The geographical distribution of this cancer was higher in the central, southern, and southwestern regions of Iran.

**Conclusion:**

The HCC incidence rate increased from 2000 to 2016, with a more significant increase in subgroups such as men, individuals over 50 years of age, and the central, southern, and southwestern regions of the country. We recommend health planners and policymakers to adopt more preventive and screening strategies for high-risk populations and provinces in Iran.

## Introduction

Primary liver cancer is the second leading cause of cancer–related deaths worldwide [1]. The dominant histologic type of liver cancer with high mortality is Hepatocellular Carcinoma (HCC) (90%), followed by intrahepatic cholangiocarcinoma (10%) [2]. The incidence of HCC is changing globally due to risk factor exposure, developing risk factor profiles, and population aging [3]. Recent increases in the incidence of this cancer can be attributed to aflatoxin exposure, lifestyle changes among urbanized populations including alcoholism, metabolic liver disease (particularly nonalcoholic fatty liver disease), obesity, and chronic hepatitis B and C [3, 4]. The Sustainable Development Goals have addressed some of these risk factors, such as viral hepatitis, alcohol usage, and obesity.

Recent data has shown an increasing rate of HCC mortality from 2.6 to 5.2 per 100,000 population during the past 20 years, globally [5]. However, there are knowledge gaps regarding HCC incidence in developing countries, mostly due to misclassifications between HCC and other primary liver cancers in the registry data. Also, there is little evidence on the trend of HCC in developing countries [1].

Iran has had a cancer registry system since 1969. The level of incompleteness and misclassification estimates of disease incidence rates in the general population are key indicators of the cancer burden. They are most often obtained by data analysis of regional, national, or international population-based cancer registries. The incidence of HCC in Iran is lower compared to other countries (annual incidence well under 5 per 100,000) [6]. To our knowledge, there weren’t any studies that had reported the incidence of HCC at national and provincial levels based on virtual data in Iran. This national and provincial study aimed to provide an overview of HCC incidence by sex, age, and geographical distribution in Iran from 2000 to 2016.

## Methods

### Overview

Data on the incidence of various cancers were extracted from the Iranian Cancer Registry and categorized based on gender, age, province of residence, nationality, and cancer type.

### Ethics statement

The Ethics Committee of the National Institute for Medical Research Development approved the study protocol (IR.NIMAD.REC.1396.192). The requirement for written informed consent was waived because of the study's retrospective nature (Registry).

#### Clarification statement

It should be mentioned that in this study, we used registry data, which is a second-hand data, and all the information was provided to us anonymous and without any ID. As a result, the privacy of patient’s data is provided.

### Dataset

Data were collected from the Iranian national program for cancer registry through the Cancer project. Data for years before 2000 were extrapolated using the random intercept-mixed effects model and the age-spatiotemporal model. The incompleteness of the cancer registry was addressed through comparison with the Social Security Insurance (SSI) organization registry. Population size and status, additional variables on wealth index, education years, and rate of urbanization was extracted from the Household Income and Expenditure Surveys, as well as Population and Housing Censuses of the Statistical Center of Iran [7].

### Data preparation

Different cancer codes based on ICD-10 and ICD-9 in the Iranian Cancer Registry system transposed into GBD codes. Classification of cancer types as defined by the International Classification of Diseases for Oncology (ICD-O) C22.0 code with the following topo-morphology codes: 8170/3, 8171/3, 8172/3, 8173/3, 8174/3, 8175/3 and 8180/3, which were used in this study. The abovementioned allocations were considered as HCC and proportioned to total liver cancer data at all levels (age, sex, year, and province). These matrix proportions were applied to liver cancer incidence data. As a result, the HCC incidence rate was determined.

#### Duplicates

This defect may be registered as a person who had been referred to or diagnosed for the first time in one province but received subsequent treatment appointments in other provinces in different years of illness. In this project, we collected data by name, surname, father's name, gender, age, and disease diagnosis. Text Mining algorithms were then used to fix this data problem so that if the similarity between the two cases was recorded up to 90%, the two circumstances were considered as one and only then included in the final calculations.

#### Misalignments

Iranian provincial divisions have changed during the last 25 years; these changes were secondary to the transfer of some cities from their original province to another province and the division of certain larger provinces into smaller ones. To fix this problem, we used information from 2011, where the country was divided into 31 provinces.

#### Incompleteness & misclassifications

The Patient’s data were recorded as topographic and morphological codes in the Iranian Cancer Registry. By applying ICD-O and based on topographic and morphological codes, all cancers were classified into 18 general categories and 70 sub-categories. In the current study and under the observation of an expert physician familiar with various disease codes, different cancer codes were extracted from ICD 10 and ICD 9. They were then matched with codes from the Global Burden of Diseases’ (GBD) cancer naming system. The collected data were categorized based on GBD codes. This procedure–in addition to removing parts of the potential inconsistency in smaller categories- allowed for a better comparison of our results with other studies.

The Social Security Organization’s (SSO) cancer registry data were used to fix the study's incompleteness. People who apply for chemotherapy drugs are covered by SSO insurance. They must also be registered in the SSO cancer registry. Consequently, it is also expected to have these people registered in the Iranian cancer registration system. The survey of people registered in SSO cancer data in different years, and those who were not registered in the Iranian cancer registry, made it possible and allowed us to obtain the Iranian cancer registry system's incompleteness rate in different years [7].

Subsequently, in a significant number of entries, the individual’s information was not fully recorded in the system. For example, the diagnosis, sex, or age of certain individuals at the time of registration was not fully documented. Furthermore, some provinces had failed to register all the cancer cases. Therefore, the data for certain times and spaces were incomplete. Different spatial and temporal locations for which certain values were not estimated were modeled using the two random intercept mixed effect and Spatio-temporal models. In summary, in the random intercept mixed effect method, a model was identified for the overall cancer incidence for each compound of age, sex, province, and year. In the next step, the first model’s residuals were redefined and modeled using the Spatio-temporal model [7].

Log(IncidenceRateofallcancer)=α+αi+log(population)+β1sex+β2age+β3year+β4YOS+β5WI+β6urbanizratio

In the final step, each cancer's contribution to overall cancer incidence was estimated using population size and Hierarchical Bootstrapping Bayesian Logistic regression model for different years. In this method, the ratios of each cancer’s incidence were calculated by the logistic regression model and the Bayesian estimation approach, in both general categories (18 cancer groups) and sub-categories. Moreover, the Bootstrap method was applied to estimate the uncertainty rate. Finally, data on the incidence of liver cancer were extracted from this series during the study period, and HCC was calculated based on the initial data proportions and evaluated.

### Statistical softwares

Statistical analysis was conducted in R programming platform and STATA software (version 14.0).

## Results

**Fig 1:**
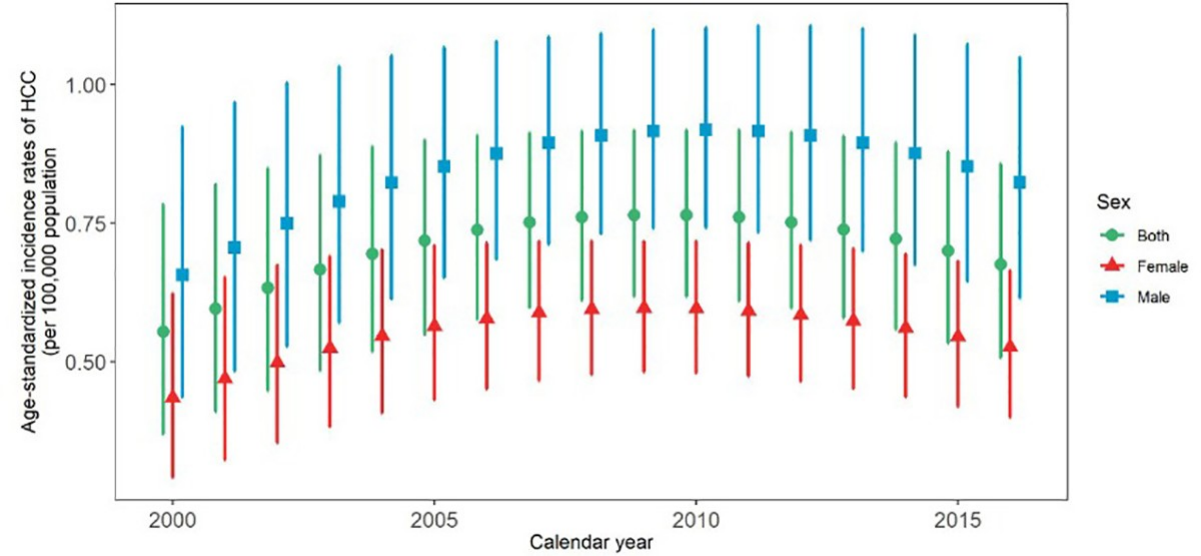
Time trend of age standardized incidence rate of HCC from 2000 to 2016.

Age-standardized incidence rate (ASIR) increased 1.17 times from 0.57 (95% UI: 0.37–0.78) per 100,000 populations in 2000 to 0.67 (0.50–0.85) in 2016 (Table 1). It should be noted, that although there had been an upward trend from 2000 to 2010, there was, however, a slight downward trend from 2010 to 2016 (Fig 1). Generally, it had a 21.89% total percentage change increase during the mentioned 17 years with 1.28 annual percentage change in both sexes (Table 1).

**Table 1:** Age standardized incidence rate of HCC in 1990 and 2016 with annual and total percent change between 1990 and 2016 at national and provincial level.

Location	2000			2016			Annual percent change			Total Percent change 2000 to 2016		
	Female	Male	Both	Female	Male	Both	Female	Male	both	Female	Male	both
National	0.44 (0.29 to 0.62)	0.66 (0.44 to 0.92)	0.55 (0.37 to 0.78)	0.53 (0.40 to 0.66)	0.82 (0.62 to 1.05)	0.68 (0.51 to 0.86)	1.23	1.50	1.29	20.92	25.52	21.89
Markazi	0.30 (0.20 to 0.42)	0.45 (0.30 to 0.63)	0.38 (0.25 to 0.53)	0.24 (0.18 to 0.30)	0.40 (0.30 to 0.50)	0.32 (0.24 to 0.40)	-1.20	-0.69	-0.98	-20.48	-11.65	-16.61
Gilan	0.18 (0.12 to 0.27)	0.28 (0.17 to 0.40)	0.23 (0.15 to 0.33)	0.15 (0.11 to 0.19)	0.22 (0.16 to 0.28)	0.18 (0.14 to 0.24)	-0.99	-1.30	-1.21	-16.78	-22.11	-20.61
Mazandaran	0.25 (0.17 to 0.37)	0.36 (0.22 to 0.52)	0.31 (0.2 0to 0.45)	0.25 (0.19 to 0.32)	0.29 (0.21 to 0.38)	0.27 (0.20 to 0.35)	-0.01	-1.06	-0.69	-0.21	-18.09	-11.72
Azarbaijan East	0.30 (0.19 to 0.45)	0.44 (0.27 to 0.65)	0.38 (0.23 to 0.55)	0.42 (0.32 to 0.53)	0.58 (0.43 to 0.74)	0.50(0.38 to 0.63)	2.39	1.93	1.99	40.58	32.74	33.79
Azarbaijan West	0.27 (0.14 to 0.47)	0.41 (0.22 to 0.67)	0.34 (0.19 to 0.58)	0.43 (0.3 to 0.58)	0.54 (0.36 to 0.74)	0.48 (0.33 to 0.66)	3.53	1.88	2.38	60	31.88	40.38
Kermnshah	0.23 (0.15 to 0.33)	0.39 (0.26 to 0.55)	0.32 (0.21 to 0.46)	0.24 (0.18 to 0.3)	0.37 (0.28 to 0.48)	0.31 (0.23 to 0.39)	0.080	-0.28	-0.32	1.30	-4.71	-5.50
Khuzestan	0.86 (0.64 to 1.1)	1.45 (1.1 to 1.82)	1.17 (0.89 to 1.48)	0.60 (0.5 to 0.71)	0.99 (0.80 to 1.18)	0.79 (0.65 to 0.95)	-1.75	-1.87	-1.89	-29.71	-31.75	-32.2
Fars	0.76 (0.55 to 0.99)	1.14 (0.84 to 1.48)	0.96 (0.71 to 1.25)	0.67 (0.55 to 0.81)	1.15 (0.92 to 1.40)	0.91 (0.73 to 1.10)	-0.63	0.08	-0.29	-10.72	1.33	-4.99
Kerman	0.91 (0.65 to 1.21)	1.51 (1.08 to 1.97)	1.23 (0.89 to 1.62)	1 (0.79 to 1.22)	1.61 (1.26 to 1.98)	1.30 (1.03 to 1.6)	0.56	0.40	0.32	9.50	6.85	5.45
Khorasan Razavi	0.38 (0.26 to 0.52)	0.57 (0.38 to 0.78)	0.48 (0.33 to 0.66)	0.44 (0.36 to 0.53)	0.70 (0.56 to 0.85)	0.57 (0.46 to 0.69)	1.05	1.33	1.10	17.90	22.64	18.69
Isfahan	0.45 (0.34 to 0.58)	0.69 (0.51 to 0.88)	0.58 (0.43 to 0.74)	0.41 (0.33 to 0.49)	0.67 (0.53 to 0.80)	0.54 (0.43 to 0.65)	-0.57	-0.17	-0.40	-9.75	-2.92	-6.87
Sistan and Baluchestan	0.26 (0.1 to 0.53)	0.40 (0.17 to 0.75)	0.33 (0.14 to 0.65)	0.40 (0.26 to 0.58)	0.70 (0.44 to 1.01)	0.55 (0.35 to 0.79)	3.30	4.50	3.78	56.15	76.49	64.33
Kurdistan	0.19 (0.09 to 0.35)	0.31 (0.15 to 0.55)	0.26 (0.12 to 0.46)	0.27 (0.16 to 0.38)	0.36 (0.21 to 0.52)	0.31 (0.19 to 0.45)	2.30	0.82	1.26	39.17	13.87	21.34
Hamedan	0.29 (0.17 to 0.43)	0.45 (0.27 to 0.67)	0.37 (0.22 to 0.56)	0.36 (0.26 to 0.47)	0.53 (0.37 to 0.71)	0.45 (0.31 to 0.59)	1.55	1.12	1.18	26.38	19.06	20.01
Chaharmahal and Bakhtiari	0.61 (0.36 to 0.95)	0.80 (0.46 to 1.21)	0.72 (0.42 to 1.1)	0.99 (0.68 to 1.32)	1.42 (0.95 to 1.92)	1.20 (0.81 to 1.62)	3.57	4.48	3.93	60.63	76.19	66.88
Lorestn	0.27 (0.16 to 0.41)	0.46 (0.28 to 0.68)	0.37 (0.23 to 0.56)	0.27 (0.2 to 0.36)	0.47 (0.33 to 0.62)	0.37 (0.26 to 0.49)	0.11	0.08	-0.05	1.90	1.38	-0.89
Ilam	0.19 (0.07 to 0.36)	0.30 (0.11 to 0.56)	0.25 (0.09 to 0.47)	0.31 (0.15 to 0.49)	0.49 (0.23 to 0.78)	0.40 (0.19 to 0.64)	3.88	3.61	3.54	66.01	61.34	60.20
Kohgiluyeh and Boyer Ahmad	0.85 (0.48 to 1.32)	1.21 (0.7 to 1.83)	1.03 (0.59 to 1.58)	1.30 (0.89 to 1.74)	1.90 (1.28 to 2.55)	1.60 (1.09 to 2.14)	3.17	3.33	3.23	53.83	56.69	54.88
Bushehr	0.32 (0.16 to 0.55)	0.38 (0.18 to 0.65)	0.35 (0.17 to 0.61)	0.22 (0.13 to 0.32)	0.28 (0.15 to 0.43)	0.26 (0.14 to 0.39)	-1.90	-1.51	-1.64	-32.31	-25.62	-27.95
Zanjan	0.07 (0.02 to 0.24)	0.12 (0.03 to 0.35)	0.10 (0.02 to 0.3)	0.08 (0.03 to 0.15)	0.10 (0.03 to 0.18)	0.09 (0.03 to 0.17)	0.71	-1.15	-0.51	12.14	-19.59	-8.67
Semnan	0.42 (0.26 to 0.6)	0.65 (0.4 to 0.93)	0.54 (0.34 to 0.78)	0.34 (0.24 to 0.45)	0.52 (0.36 to 0.7)	0.43 (0.3 to 0.58)	-1.08	-1.10	-1.17	-18.30	-18.72	-19.84
Yazd	1.63 (1.19 to 2.1)	2.21 (1.62 to 2.84)	1.98 (1.45 to 2.53)	2.22 (1.78 to 2.67)	2.42 (1.91 to 2.94)	2.32 (1.85 to 2.81)	2.14	0.55	1.02	36.46	9.29	17.38
Hormozgan	0.28 (0.1 to 0.66)	0.41 (0.16 to 0.89)	0.35 (0.13 to 0.79)	0.30 (0.16 to 0.50)	0.61 (0.31 to 0.99)	0.46 (0.23 to 0.75)	0.46	2.88	1.78	7.87	48.99	30.34
Tehran	0.45 (0.32 to 0.62)	0.65 (0.44 to 0.89)	0.55 (0.38 to 0.76)	0.55 (0.43 to 0.67)	1.030 (0.79 to 1.27)	0.78 (0.61 to 0.97)	1.28	3.45	2.47	21.80	58.60	42.07
Ardabil	0.18 (0.09 to 0.31)	0.26 (0.13 to 0.43)	0.22 (0.11 to 0.38)	0.20 (0.13 to 0.27)	0.25 (0.16 to 0.36)	0.23 (0.14 to 0.31)	0.63	-0.11	0.09	10.66	-1.91	1.45
Qom	0.18 (0.09 to 0.31)	0.24 (0.11 to 0.41)	0.21 (0.1 to 0.36)	0.26 (0.16 to 0.36)	0.38 (0.23 to 0.54)	0.32 (0.20 to 0.45)	2.62	3.53	3.09	44.48	59.98	52.57
Qazvin	0.16 (0.07 to 0.30)	0.24 (0.10 to 0.44)	0.20 (0.09 to 0.37)	0.27 (0.16 to 0.39)	0.44 (0.25 to 0.65)	0.35 (0.20 to 0.52)	3.75	5.03	4.40	63.78	85.47	74.78
Golestan	0.22 (0.11 to 0.41)	0.33 (0.17 to 0.59)	0.28 (0.14 to 0.50)	0.35 (0.23 to 0.50)	0.49 (0.29 to 0.71)	0.42 (0.26 to 0.61)	3.68	2.69	2.92	62.58	45.78	49.56
Khorasan North	0.22 (0.09 to 0.42)	0.35 (0.14 to 0.64)	0.29 (0.12 to 0.54)	0.30 (0.17 to 0.45)	0.47 (0.26 to 0.7)	0.38 (0.21 to 0.58)	2.20	2.09	2.01	37.41	35.49	34.18
Khorasan South	0.17 (0.06 to 0.34)	0.28 (0.10 to 0.53)	0.23 (0.08 to 0.44)	0.18 (0.09 to 0.28)	0.32 (0.16 to 0.5)	0.25 (0.12 to 0.39)	0.43	0.91	0.64	7.27	15.45	10.93
Alborz	0.87 (0.59 to 1.25)	1.05 (0.68 to 1.52)	0.97 (0.63 to 1.39)	1.83 (1.4 to 2.3)	2.75 (2.08 to 3.48)	2.29 (1.74 to 2.89)	6.44	9.46	8.04	109.51	160.83	136.66

Male (0.65: 0.43–0.92, per 100,000 population) to female (0.43: 0.29–0.62) ASIR ratio in 2000 was 1.51. In 2016, male (0.82: 0.61–1.04) to female (0.52: 0.40–0.66) ASIR ratio was 1.57. The male and female annual percentage change was 1.50 and 1.23, respectively (Table 1).

**Fig 2:**
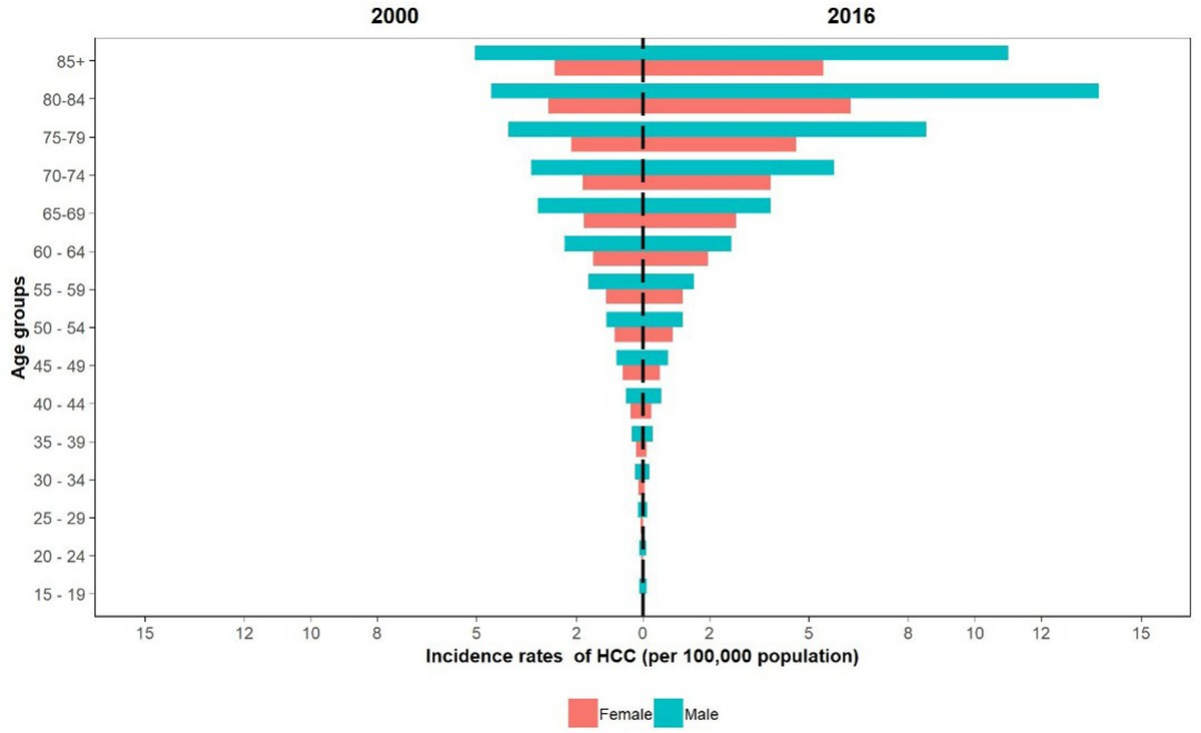
Comparison of incidence rate of HCC by age group and sex, 2000 vs 2016.

Overall, after the age of 50 years, the incidence of HCC increased dramatically with age and increased from 1.19 (0.98–1.40) in the 50–55 age group to 6.65 (5.45–7.78) in the >85 age group. Despite the rising pattern, the incidence was lower than one per 100000 people in the <50 age group (Fig 2).

**Fig 3:**
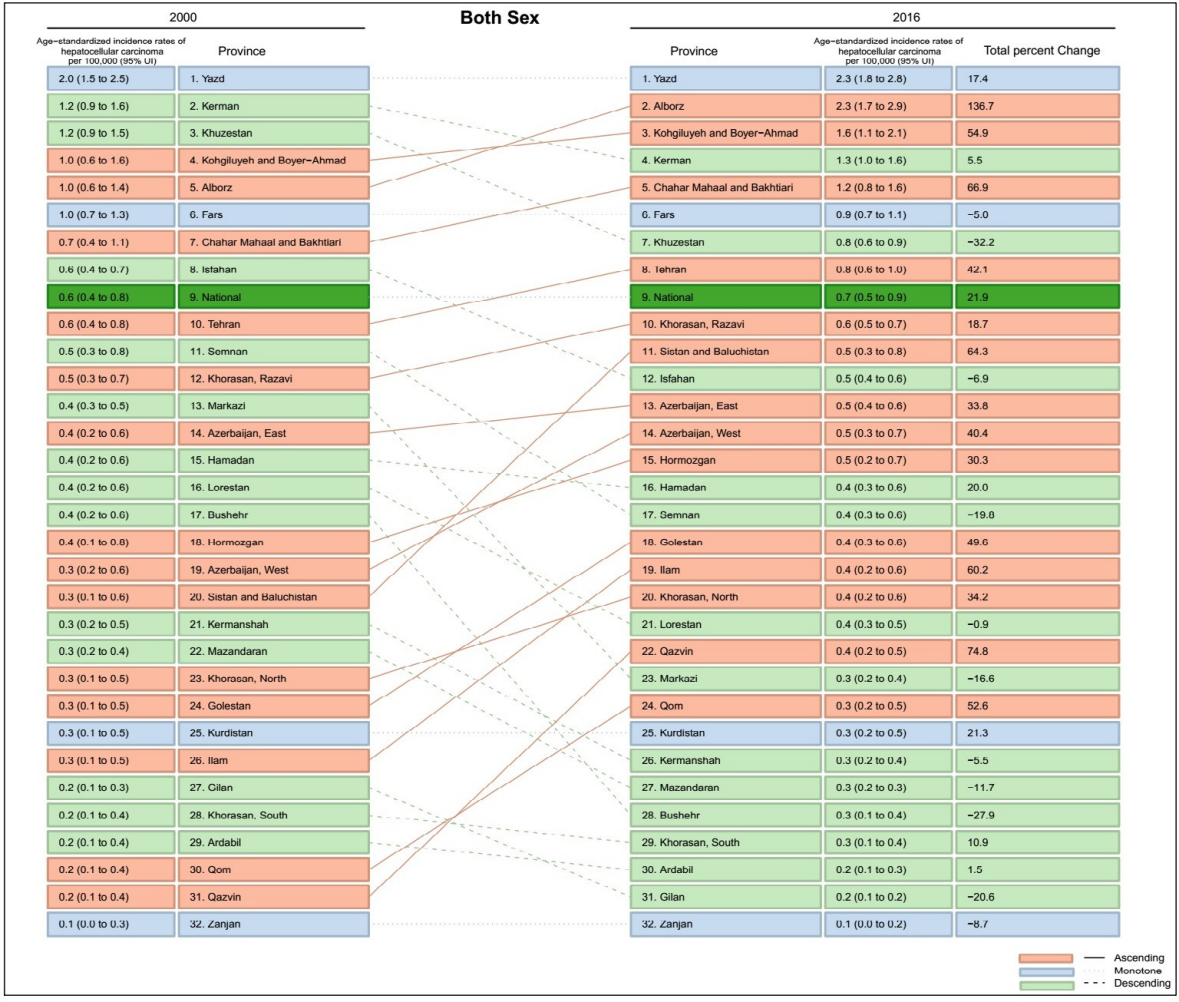
Comparison of age standardized incidence rate of HCC in different provinces, 2000 vs 2016.

**Fig 4:**
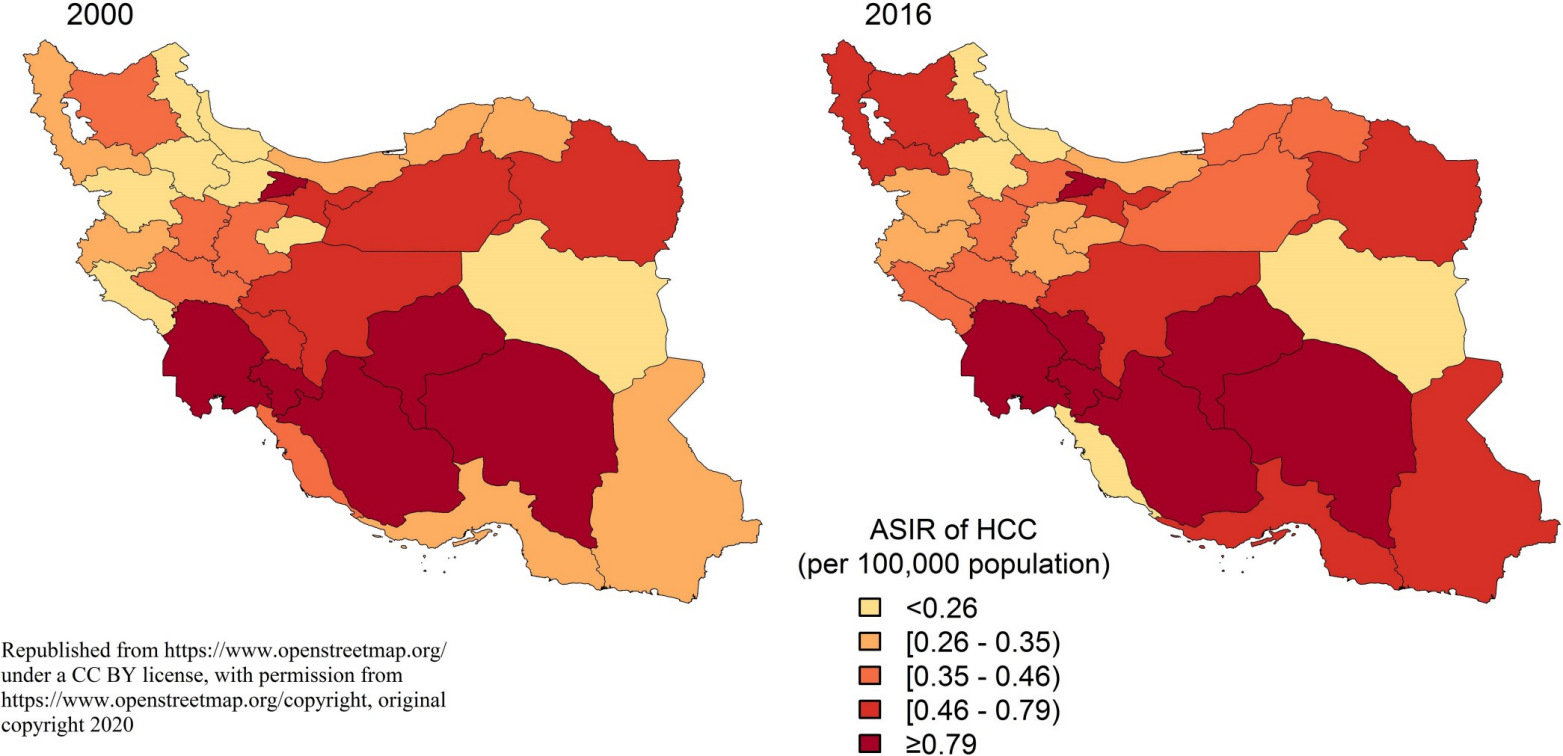
Geographical distribution of HCC in different provinces of Iran, 2000 vs 2016 [8].

At the provincial level, the highest to lowest incidence rate ratio among provinces in 2000 was 21.8. Moreover, the highest to lowest proportion was 28.8 in 2016 (Figs 3 and 4). The ASIR ratio of HCC in all provinces of the country was higher among males in both 2000 and 2016 (Table 1).

During the study period, 11 provinces had a negative total percentage change of HCC ASIR. In other words, the ASIR in these provinces at the end of the study period was lower than it was at the beginning of the study. The highest decrease was -31%. Conversely, the other 21 provinces had an increased ASIR at the end of the study. The highest total percentage change was 137% (Fig 3).

Evaluation of the Annual Percent Change (APC) of ASIR showed that the lowest and highest APCs of ASIR were -1.89 and +8.04, respectively. Also, in both 2000 and 2016, ASIR was higher among males in all provinces (Table 1).

## Discussion

Based on our results, the incidence of HCC had witnessed a downward trend since 2010. However, the incidence rate increased over the study period. The geographical distribution of this cancer had always been higher in the central, southern and southwestern regions of Iran. The highest to lowest incidence rate ratio among provinces was 21.8 in 2000 and 28.8 in 2016. In terms of gender, HCC incidence rate was always 1.5 times higher in males. It should be noted that the incidence rate increased dramatically from the age of 50–55 years.

The increased incidence of liver cancer had been confirmed in other studies from the US, Europe, and Australia. Various US studies often cited the increasing incidence of liver cancer during the past three decades until 2007, which later followed a downward trend [9, 10]. Another study in Japan, which looked at mortality from liver cancer and hepatocellular carcinoma in Japan, found that deaths from the disease had risen sharply since 1975. It began to decline since 2002 [11]. Another study in Shanghai examining the incidence of hepatocellular carcinoma during 1976–2005 found that the incidence of the disease had decreased in both sexes [12]. However, some studies from the United States showed that HCC incidence was rising by 2012 [13]. Two different Australian studies, one in 2010 and another in 2014, reported an upward trend in cancer incidence and did not report any decline [1, 14]. Another study in Denmark reported an increasing trend from 1994 to 2016, with no decrease [15]. Various studies from Iran showed a rising trend of this cancer from 2003 to 2012 [16, 17]. In our study, there was an upward trend until 2010, followed by a downward trend. Therefore, unlike this study or the one conducted in the United States, liver cancer incidence continued to rise and no decline was reported in either Australia or Denmark. Various causes have been reported to justify the rising trend of the disease. The most important are viral hepatitis, especially hepatitis B and C, the increasing trend of obesity and metabolic syndrome in all age groups in developed and developing countries, aflatoxin exposure, and alcohol. The increased access to and expansion of diagnostic tools, changing diagnostic criteria for HCC, and increased life expectancy and population aging are other contributors to elevated HCC incidence [6, 13, 16, 18]. Although there are several causes of the disease, various studies in the US, Europe, Australia, and elsewhere have identified hepatitis B and C as significant causes of HCC [14, 18]. The decreasing trend in China and Japan is mainly attributed to the initiation of hepatitis B vaccination and hepatitis C interferon therapy [11, 12]. Albeit low in prevalence, Hepatitis B is the leading cause of HCC in the world, and the best way to combat it is vaccination, which has begun some 20 years ago. However, since most affected patients are over 50 years of age, and vaccination has not been observed in some countries, it takes longer to show its effect. But hepatitis C does not have a vaccine. The only available precautionary measure is to fight risky behaviors and perform the better screening during blood transfusions [14, 18]. However, it should be noted that hepatitis B is not the only cause of HCC. Some studies believe that the leading cause of HCC is shifting from hepatitis B to hepatitis C, alcohol, cirrhosis, and non-alcoholic fatty liver disease (NAFLD) [13]. Importantly, NAFLD is on the rise and unlike hepatitis and alcohol, it can lead to cancer without cirrhosis [1, 13, 14, 19, 20]. Other contributors to the rising trend in cancer can include increased access to diagnostic, imaging, and testing facilities. By strengthening these possibilities, many cases that had previously died without a diagnosis were diagnosed, and it led to the increased incidence of disease [9, 15]. Since the highest incidence is seen in older age groups, increased life expectancy and population aging may also cause increasing trend in cancer [16].

Contrary to the Australian and Danish studies, our study showed that the incidence rate had declined from a certain point in time, which was compatible with US studies. This could be due to viral hepatitis screening done before blood transfusions and the subsequently increased detection and treatment of hepatitis C. Another factor may be hepatitis B vaccination, which began in 1994 in Iran, which has impacted certain countries over time [10].

The Geographical disparity in HCC incidence rates had been observed in studies of other countries. The primary cause of these geographic differences is due to differences in the rates of exposure to viral hepatitis and other liver cancer risk factors [21], which include obesity, diabetes, metabolic syndrome, alcohol abuse, cirrhosis, NAFLD and exposure to aflatoxin [1, 15, 16, 21]. Pistachio, one of Iran’s most afmous products mainly cultivated in central provinces, is a source of aflatoxin. Kerman is a province in Iran that has consistently had one of the highest HCC rates in various studies. The province is the largest producer of pistachios, and studies have reported that 36.7% of Iranian pistachios are contaminated with aflatoxins [6]. Another study in Semnan province, revealed an ASIR of 5.83 and 3.53 per 100,000 population among men and women, respectively. These were the highest HCC rates in Iran, attributable to hepatitis B, C, and liver cirrhosis [16].

Another important finding of this study was the male to female HCC incidence ratio. According to a Danish study, the male to female ratio was 3 to 1 [15]. It is worth noting that this ratio increased with age. In our study, this difference was most prominent in the >80 age group. In another study in the United States, this proportion increased with age and reached its highest level in the 50–64 age group [13]. Given the disparity between the two sexes, estrogen and androgens are thought to regulate liver carcinogenesis [11].

Ninety-five percent of HCC cases in the Australian and American studies were over 45 years of age, and the highest incidence was seen in the US study among 55-64-year-olds [1, 13]. In the Danish study, the highest rates were found among 75-79-year-old males and 80-84-year-old females [15]. In Spain, there was a 4-fold increase in liver cancer incidence in people over the age of 54 [11].

One of the advantages of the present study was that it reported results for the whole country. Another benefit was addressing the incompleteness of the Iranian cancer registry system data using the SSO cancer registry data and statistical methods. Moreover, to the best of our knowledge, epidemiologic indices of HCC in all age and sex groups and provincial levels throughout the study period were available (except inadequate information in certain regions). We extracted the HCC incidence rate from total liver cancer incidence rates in all the above-mentioned details at national and provincial levels for both sexes and all age groups. One of the limitations of this study was the lack of data on the staging of liver cancer. Another limitation was the lack of data and information on the risk factors of liver cancer, its prognosis, and its surveillance in Iran. Therefore, we suggest other researchers work on the mortality rates of different stages of the disease, prognosis, and surveillance rates in Iran.

## Conclusion

HCC incidence rate had experienced an upward trend during the past decades, but it declined in recent years. This reduction in the incidence of the disease may suggest a role for hepatitis B vaccination. Due to the high incidence and poor prognosis of this disease, it is necessary to conduct more epidemiological studies to identify other risk factors and also to monitor the effect of continued vaccination to control this disease. There was a geographical difference in HCC incidence, with higher incidence rates in central, southern, and southwestern parts of the country. The statistics presented in this article about the differences between provinces, both sexes and disease trends can be reliable resources for health planners in the country to design and implement appropriate programs to continue the downward trend.

## Supporting information

S1 Data (XLS)
